# Diaqua­tris­[4,4,4-trifluoro-3-oxo-1-(thio­phen-2-yl)but-1-en-1-olato]neodymium(III) acetonitrile monosolvate

**DOI:** 10.1107/S1600536811030376

**Published:** 2011-08-02

**Authors:** Patrick S. Barber, Ana de Bettencourt-Dias

**Affiliations:** a University of Nevada, Reno, NV 89557, USA

## Abstract

The title complex, [Nd(C_8_H_4_F_3_O_2_S)_3_(H_2_O)_2_]·CH_3_CN, consists of an Nd^III^ ion surrounded by three 4,4,4-trifluoro-3-oxo-1-(thio­phen-2-yl)but-1-en-1-olate ligands, coordinated through the O atoms, and two water mol­ecules. The Nd—O bond lengths range from 2.372 (2) to 2.513 (2) Å. The metal ion displays a coordination number of eight and a square-anti­prismatic coordination geometry. A single uncoordinated acetonitrile mol­ecule is present in the asymmetric unit. Two of the three thio­phene rings are disordered, resulting from a 180° rotation with respect to the β-diketonate moiety. The coordinated water mol­ecules act as hydrogen-bond donors towards the acetonitrile N atom and the β-diketonate O atoms.

## Related literature

The title complex has been studied for its near-infrared emitting properties and as a standard for near-infrared emission quantum yield measurements, see: Rusakova *et al.* (1992*a* ▶,*b* ▶); Voloshin *et al.* (2000 ▶). For the Eu^III^ analog, see: White (1976 ▶). A similar Nd^III^ complex with trifluoroacetyl-4-(thio­phen2-yl)acetonato ligands but with triphenyl­phosphine oxide instead of water mol­ecules as the ancillary ligands was described by Leipoldt *et al.* (1975 ▶).
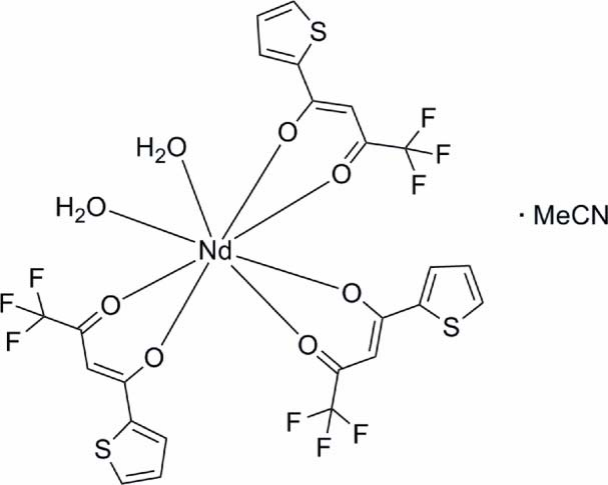


## Experimental

### 

#### Crystal data

[Nd(C_8_H_4_F_3_O_2_S)_3_(H_2_O)_2_]·C_2_H_3_N
*M* *_r_* = 884.84
Triclinic, 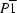

*a* = 11.2381 (1) Å
*b* = 12.5467 (1) Å
*c* = 13.3618 (1) Å
α = 66.038 (1)°
β = 68.586 (1)°
γ = 71.955 (1)°
*V* = 1573.66 (2) Å^3^
*Z* = 2
Mo *K*α radiation
μ = 1.95 mm^−1^
*T* = 100 K
0.15 × 0.08 × 0.03 mm

#### Data collection

Bruker APEX CCD diffractometer
Absorption correction: multi-scan (*SADABS*; Sheldrick, 2008 ▶) *T*_min_ = 0.759, *T*_max_ = 0.953
43274 measured reflections
9148 independent reflections
7549 reflections with *I* > 2σ(*I*)
*R*_int_ = 0.054

#### Refinement

*R*[*F* ^2^ > 2σ(*F* ^2^)] = 0.035
*wR*(*F* ^2^) = 0.088
*S* = 1.10
9148 reflections
424 parameters
20 restraints
H-atom parameters constrained
Δρ_max_ = 0.96 e Å^−3^
Δρ_min_ = −1.06 e Å^−3^

### 

Data collection: *SMART* (Bruker, 2001 ▶); cell refinement: *SAINT* (Bruker, 2001 ▶); data reduction: *SAINT*; program(s) used to solve structure: *SHELXS97* (Sheldrick, 2008 ▶); program(s) used to refine structure: *SHELXL97* (Sheldrick, 2008 ▶); molecular graphics: *DIAMOND* (Brandenburg, 1999 ▶); software used to prepare material for publication: *SHELXTL* (Sheldrick, 2008 ▶).

## Supplementary Material

Crystal structure: contains datablock(s) global. DOI: 10.1107/S1600536811030376/im2307sup1.cif

Additional supplementary materials:  crystallographic information; 3D view; checkCIF report

## Figures and Tables

**Table 1:** Hydrogen-bond geometry (Å, °)

*D*—H⋯*A*	*D*—H	H⋯*A*	*D*⋯*A*	*D*—H⋯*A*
O7—H7*A*⋯O5^i^	0.84	2.02	2.811 (3)	156
O7—H7*B*⋯O4^i^	0.85	2.06	2.812 (3)	148
O8—H8*A*⋯N1	0.84	2.15	2.902 (5)	149

Symmetry code: (i) 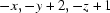.

**Fig. 1.**
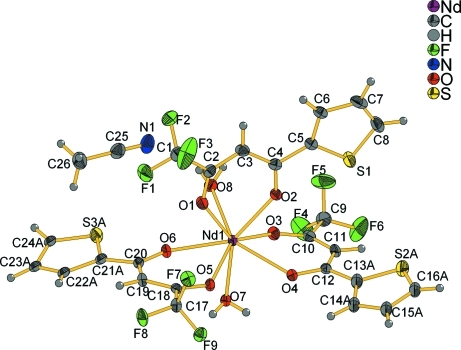
Thermal ellipsoid plot of the title complex with atom numbering (except hydrogen atoms) and 50% probability displacement ellipsoids for non-hydrogen atoms. Only the major component of the disordered thiophene rings is shown.

**Fig. 2.**
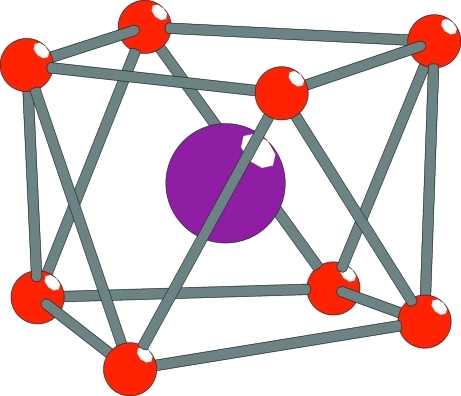
Coordination polyhedron around the NdIII showing a slightly distorted square antiprismatic geometry.

**Fig. 3.**
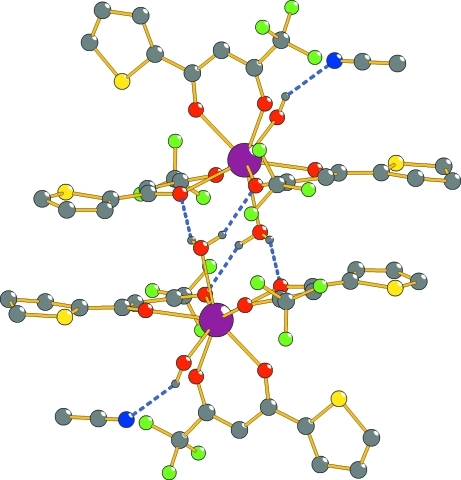
Ball-and-stick representation showing packing with hydrogen bonding interactions as dashed blue lines. Hydrogen atoms not involved in these interactions were omitted for clarity.

## supplementary crystallographic information

### Comment

For determining emission efficiencies of sensitized lanthanide luminescence in the NIR region of the spectrum, a Nd^III^ complex with 4,4,4-trifluoro-3-oxo-1-(thiophen-2-yl)but-1-en-1-olate (TTA) (Scheme 1) is used as a standard (Rusakova *et al.*, 1992*a*). We synthesized the complex and isolated X-ray quality single crystals. The structure shown in Figure 1 was obtained. The Nd^III^ is surrounded by three TTA ligands, coordinated through the oxygen atoms, and two water molecules. Two TTA ligands are in the same plane while the remaining one is almost perpendicular to that plane with an angle of 87.6°. A single uncoordinated acetonitrile molecule crystallized within the asymmetric unit. Two of the three thiophene rings are disordered, as seen for many structures involving thiophene moieties. Modeling the disorder gives two different positions for the thiophene rings, where they are rotated 180° around the C—C bond to the beta-diketone. The occupancy factors for both disordered rings are 59 and 61% for the major component. Figure 2 shows the coordination polyhedron around the Nd^III^, which can be described as a slightly distorted square antiprism. The Nd^III^—O bond distances are in the range 2.372 (3)–2.513 (3) Å with the TTA ligands displaying the shorter bond lengths. These distances compare well with a similar structure of Nd^III^ with TTA reported by Leipoldt *et al.* (1975), with the formula [Nd(TTA)_3_(TPPO)_2_] (TPPO=triphenylphosphine oxide). In this complex the two water molecules are replaced by two TPPO ligands. The final complex has a similar coordination environment around the Nd^III^ ion with Nd^III^—O bond distances in the range 2.397–2.496 Å, with the shorter bond distances corresponding to the TPPO ligands.

Hydrogen bonding interactions support the packing structure of the complex and are shown in Figure 3 with blue dashed lines. Interactions exist between the water molecule O—H donors and the acetonitrile N and beta-diketonato O acceptors with D···A distances in the range 2.803 (3)–2.893 (4) Å. A search of similar interactions in the Cambridge Structural Database yields 194 results with a range of interaction distances between 2.597 and 3.040 Å and an average of 2.834 Å. The reported distances are slightly longer than the distances in the complex discussed here. An isostructural Eu(III) complex has also been reported by White (1976). Due to slow decomposition of the crystals of the Eu(III) complex the data reported were poor, but the weak hydrogen bonding interactions were shown to be in the range D···A = 2.9–3.0 Å.

### Experimental

The title compound was synthesized by a previously reported procedure (Voloshin *et al.* 2000). Crystals were isolated from a saturated solution of acetonitrile and water (*v/v* = 1:5) upon standing at room temperature.

### Refinement

Hydrogen atoms were positioned geometrically using a riding model with C—H = 0.95, 0.99 and 0.98 Å for aromatic CH and aliphatic CH_2_ and CH_3_ hydrogen atoms, respectively, and U_iso_(H)=1.2–1.5 U_eq_(C). Hydrogen atoms of the water molecules were found in the difference map and their thermal parameters constrained to the parent atoms. Two thiophene rings are disordered through a 180° rotation with respect to the C—C bond to the β-diketonato moiety. The disorder was modeled by allowing the occupancy factors of the two possible ring positions to freely refine to 61 and 59% for the major components. The bond distances for the major and minor components were restrained with the SAME command and the thermal displacement paramenters of both ring atom components restrained with the EADP command.

### Crystal data

**Table:**

[Nd(C_8_H_4_F_3_O_2_S)_3_(H_2_O)_2_]·C_2_H_3_N	*Z* = 2
*M**_r_* = 884.84	*F*(000) = 870
Triclinic, *P*1	*D*_x_ = 1.867 Mg m^−^^3^
Hall symbol: -P 1	Mo *K*α radiation, λ = 0.71073 Å
*a* = 11.2381 (1) Å	Cell parameters from 9284 reflections
*b* = 12.5467 (1) Å	θ = 2.3–31.3°
*c* = 13.3618 (1) Å	µ = 1.95 mm^−^^1^
α = 66.038 (1)°	*T* = 100 K
β = 68.586 (1)°	Plates, colourless
γ = 71.955 (1)°	0.15 × 0.08 × 0.03 mm
*V* = 1573.66 (2) Å^3^	

### Data collection

**Table:**

Bruker APEX CCD diffractometer	9148 independent reflections
Radiation source: fine-focus sealed tube	7549 reflections with *I* > 2σ(*I*)
graphite	*R*_int_ = 0.054
φ and ω scans	θ_max_ = 30.0°, θ_min_ = 1.7°
Absorption correction: multi-scan (*SADABS*; Sheldrick, 2008)	*h* = −15→15
*T*_min_ = 0.759, *T*_max_ = 0.953	*k* = −17→17
43274 measured reflections	*l* = −18→18

### Refinement

**Table:**

Refinement on *F*^2^	Primary atom site location: structure-invariant direct methods
Least-squares matrix: full	Secondary atom site location: difference Fourier map
*R*[*F*^2^ > 2σ(*F*^2^)] = 0.035	Hydrogen site location: inferred from neighbouring sites
*wR*(*F*^2^) = 0.088	H-atom parameters constrained
*S* = 1.10	*w* = 1/[σ^2^(*F*_o_^2^) + (0.044*P*)^2^] where *P* = (*F*_o_^2^ + 2*F*_c_^2^)/3
9148 reflections	(Δ/σ)_max_ = 0.007
424 parameters	Δρ_max_ = 0.96 e Å^−^^3^
20 restraints	Δρ_min_ = −1.06 e Å^−^^3^

### Special details

**Table:**

Geometry. All e.s.d.'s (except the e.s.d. in the dihedral angle between two l.s. planes) are estimated using the full covariance matrix. The cell e.s.d.'s are taken into account individually in the estimation of e.s.d.'s in distances, angles and torsion angles; correlations between e.s.d.'s in cell parameters are only used when they are defined by crystal symmetry. An approximate (isotropic) treatment of cell e.s.d.'s is used for estimating e.s.d.'s involving l.s. planes.
Refinement. Refinement of *F*^2^ against ALL reflections. The weighted *R*-factor *wR* and goodness of fit *S* are based on *F*^2^, conventional *R*-factors *R* are based on *F*, with *F* set to zero for negative *F*^2^. The threshold expression of *F*^2^ > σ(*F*^2^) is used only for calculating *R*-factors(gt) *etc*. and is not relevant to the choice of reflections for refinement. *R*-factors based on *F*^2^ are statistically about twice as large as those based on *F*, and *R*- factors based on ALL data will be even larger.

### Fractional atomic coordinates and isotropic or equivalent isotropic displacement parameters (Å^2^)

**Table:**

	*x*	*y*	*z*	*U*_iso_*/*U*_eq_	Occ. (<1)
Nd1	0.190944 (16)	0.993398 (14)	0.285170 (14)	0.01360 (5)	
C1	0.3737 (4)	0.6939 (3)	0.1120 (3)	0.0285 (8)	
C2	0.2963 (3)	0.8164 (3)	0.1216 (3)	0.0196 (6)	
C3	0.2577 (3)	0.8976 (3)	0.0273 (3)	0.0223 (7)	
H3	0.2875	0.8788	−0.0410	0.027*	
C4	0.1747 (3)	1.0083 (3)	0.0291 (3)	0.0183 (6)	
C5	0.1205 (3)	1.0837 (3)	−0.0673 (3)	0.0185 (6)	
C6	0.1273 (3)	1.0597 (3)	−0.1632 (3)	0.0202 (6)	
H6	0.1714	0.9876	−0.1783	0.024*	
C7	0.0588 (4)	1.1585 (4)	−0.2347 (3)	0.0300 (8)	
H7	0.0520	1.1594	−0.3038	0.036*	
C8	0.0047 (4)	1.2505 (3)	−0.1950 (3)	0.0291 (8)	
H8	−0.0428	1.3234	−0.2336	0.035*	
C9	0.1372 (4)	1.4073 (3)	0.1154 (3)	0.0292 (8)	
C10	0.0882 (3)	1.2891 (3)	0.1831 (3)	0.0198 (6)	
C11	−0.0394 (3)	1.2887 (3)	0.1983 (3)	0.0220 (7)	
H11	−0.0910	1.3607	0.1633	0.026*	
C12	−0.0985 (3)	1.1890 (3)	0.2622 (3)	0.0175 (6)	
C17	0.0664 (3)	0.6343 (3)	0.5390 (3)	0.0191 (6)	
C18	0.1538 (3)	0.7266 (3)	0.4876 (2)	0.0152 (6)	
C19	0.2779 (3)	0.6892 (3)	0.4989 (3)	0.0178 (6)	
H19	0.3035	0.6083	0.5409	0.021*	
C20	0.3703 (3)	0.7649 (3)	0.4512 (3)	0.0161 (6)	
C25	0.6428 (4)	0.7441 (3)	0.1595 (3)	0.0305 (8)	
C26	0.6983 (4)	0.6270 (4)	0.2297 (4)	0.0400 (10)	
H26A	0.6717	0.6243	0.3090	0.060*	
H26B	0.7934	0.6137	0.2015	0.060*	
H26C	0.6665	0.5650	0.2253	0.060*	
F1	0.4227 (3)	0.62708 (19)	0.1994 (2)	0.0422 (6)	
F2	0.4754 (2)	0.7038 (2)	0.0187 (2)	0.0433 (6)	
F3	0.3014 (3)	0.6348 (2)	0.1032 (3)	0.0691 (10)	
F4	0.2109 (3)	1.4231 (2)	0.1633 (2)	0.0566 (8)	
F5	0.2082 (3)	1.4097 (2)	0.0104 (2)	0.0528 (7)	
F6	0.0425 (3)	1.5023 (2)	0.1042 (3)	0.0606 (8)	
F7	0.0536 (2)	0.60903 (17)	0.45471 (17)	0.0257 (4)	
F8	0.1102 (2)	0.53106 (17)	0.61107 (17)	0.0273 (4)	
F9	−0.05393 (18)	0.67559 (17)	0.59475 (17)	0.0236 (4)	
N1	0.5971 (3)	0.8339 (3)	0.1063 (3)	0.0356 (8)	
O1	0.2734 (2)	0.8264 (2)	0.21758 (19)	0.0210 (5)	
O2	0.1425 (2)	1.04824 (19)	0.11022 (18)	0.0181 (4)	
O3	0.1768 (2)	1.20437 (19)	0.21461 (19)	0.0204 (5)	
O4	−0.0423 (2)	1.08892 (19)	0.31882 (18)	0.0176 (4)	
O5	0.0960 (2)	0.83027 (18)	0.43937 (18)	0.0171 (4)	
O6	0.3515 (2)	0.87030 (19)	0.38596 (18)	0.0179 (4)	
O7	0.1452 (2)	1.05215 (19)	0.45633 (18)	0.0180 (4)	
H7A	0.0702	1.0939	0.4681	0.027*	
H7B	0.1300	0.9887	0.5114	0.027*	
O8	0.4046 (2)	1.0376 (2)	0.1502 (2)	0.0244 (5)	
H8A	0.4422	0.9834	0.1213	0.037*	
H8B	0.4229	1.0902	0.0844	0.037*	
S1	0.03086 (9)	1.22193 (8)	−0.06754 (8)	0.02789 (19)	
S2A	−0.3133 (2)	1.31625 (18)	0.1803 (2)	0.0320 (5)	0.594 (3)
C13A	−0.2345 (3)	1.1990 (3)	0.2668 (3)	0.0242 (5)	0.594 (3)
C14A	−0.3198 (9)	1.1137 (9)	0.3392 (8)	0.0242 (5)	0.594 (3)
H14A	−0.2951	1.0439	0.3974	0.029*	0.594 (3)
C15A	−0.4361 (7)	1.1402 (6)	0.3183 (6)	0.0242 (5)	0.594 (3)
H15A	−0.5016	1.0931	0.3586	0.029*	0.594 (3)
C16A	−0.4458 (7)	1.2445 (5)	0.2311 (6)	0.0242 (5)	0.594 (3)
H16A	−0.5182	1.2759	0.2002	0.029*	0.594 (3)
S2B	−0.3167 (4)	1.0888 (3)	0.3546 (3)	0.0320 (5)	0.406 (3)
C13B	−0.2345 (3)	1.1990 (3)	0.2668 (3)	0.0242 (5)	0.406 (3)
C14B	−0.3102 (13)	1.2881 (12)	0.1790 (13)	0.0242 (5)	0.406 (3)
H14B	−0.2735	1.3422	0.1076	0.029*	0.406 (3)
C15B	−0.4371 (10)	1.2763 (8)	0.2204 (9)	0.0242 (5)	0.406 (3)
H15B	−0.5072	1.3359	0.1971	0.029*	0.406 (3)
C16B	−0.4491 (10)	1.1696 (8)	0.2981 (9)	0.0242 (5)	0.406 (3)
H16B	−0.5257	1.1386	0.3218	0.029*	0.406 (3)
S3A	0.60840 (19)	0.8046 (2)	0.41395 (14)	0.0263 (4)	0.609 (3)
C21A	0.4947 (3)	0.7201 (3)	0.4798 (3)	0.0223 (5)	0.609 (3)
C22A	0.5414 (9)	0.6050 (10)	0.5596 (8)	0.0223 (5)	0.609 (3)
H22A	0.4990	0.5387	0.5975	0.027*	0.609 (3)
C23A	0.6670 (7)	0.6111 (6)	0.5706 (5)	0.0223 (5)	0.609 (3)
H23A	0.7069	0.5592	0.6293	0.027*	0.609 (3)
C24A	0.7138 (6)	0.7048 (6)	0.4809 (5)	0.0223 (5)	0.609 (3)
H24A	0.8023	0.7120	0.4585	0.027*	0.609 (3)
S3B	0.5321 (4)	0.5908 (4)	0.5775 (3)	0.0263 (4)	0.391 (3)
C21B	0.4947 (3)	0.7201 (3)	0.4798 (3)	0.0223 (5)	0.391 (3)
C22B	0.5922 (13)	0.7958 (14)	0.4398 (12)	0.0223 (5)	0.391 (3)
H22B	0.5893	0.8744	0.3871	0.027*	0.391 (3)
C23B	0.6955 (10)	0.7251 (8)	0.4981 (8)	0.0223 (5)	0.391 (3)
H23B	0.7625	0.7536	0.5016	0.027*	0.391 (3)
C24B	0.6742 (11)	0.6036 (9)	0.5488 (8)	0.0223 (5)	0.391 (3)
H24B	0.7424	0.5371	0.5633	0.027*	0.391 (3)

### Atomic displacement parameters (Å^2^)

**Table:**

	*U*^11^	*U*^22^	*U*^33^	*U*^12^	*U*^13^	*U*^23^
Nd1	0.01429 (8)	0.01215 (8)	0.01391 (8)	−0.00136 (5)	−0.00544 (6)	−0.00335 (6)
C1	0.031 (2)	0.0238 (17)	0.0302 (19)	0.0008 (15)	−0.0090 (16)	−0.0128 (15)
C2	0.0183 (16)	0.0162 (14)	0.0239 (16)	−0.0033 (12)	−0.0036 (13)	−0.0082 (13)
C3	0.0289 (18)	0.0188 (15)	0.0182 (15)	−0.0048 (13)	−0.0041 (13)	−0.0072 (13)
C4	0.0180 (15)	0.0214 (15)	0.0174 (14)	−0.0083 (12)	−0.0035 (12)	−0.0063 (13)
C5	0.0204 (16)	0.0189 (15)	0.0173 (14)	−0.0076 (12)	−0.0051 (12)	−0.0045 (12)
C6	0.0222 (16)	0.0248 (16)	0.0158 (14)	−0.0115 (13)	−0.0050 (13)	−0.0038 (13)
C7	0.0282 (19)	0.048 (2)	0.0167 (16)	−0.0194 (17)	−0.0055 (14)	−0.0059 (16)
C8	0.0270 (19)	0.035 (2)	0.0245 (17)	−0.0097 (16)	−0.0143 (15)	−0.0001 (16)
C9	0.034 (2)	0.0186 (16)	0.0285 (18)	−0.0044 (15)	−0.0047 (16)	−0.0048 (15)
C10	0.0289 (18)	0.0140 (14)	0.0155 (14)	−0.0029 (13)	−0.0053 (13)	−0.0054 (12)
C11	0.0274 (18)	0.0148 (14)	0.0179 (15)	0.0028 (13)	−0.0091 (13)	−0.0020 (12)
C12	0.0192 (15)	0.0194 (15)	0.0147 (14)	0.0010 (12)	−0.0065 (12)	−0.0082 (12)
C17	0.0201 (16)	0.0155 (14)	0.0236 (16)	−0.0009 (12)	−0.0107 (13)	−0.0060 (13)
C18	0.0174 (15)	0.0153 (14)	0.0128 (13)	−0.0025 (11)	−0.0046 (11)	−0.0046 (11)
C19	0.0180 (15)	0.0151 (14)	0.0214 (15)	−0.0010 (12)	−0.0105 (13)	−0.0042 (12)
C20	0.0157 (15)	0.0174 (14)	0.0158 (14)	0.0015 (11)	−0.0059 (12)	−0.0080 (12)
C25	0.0227 (18)	0.032 (2)	0.034 (2)	−0.0063 (15)	−0.0050 (16)	−0.0097 (17)
C26	0.034 (2)	0.035 (2)	0.034 (2)	−0.0024 (18)	−0.0093 (18)	0.0028 (18)
F1	0.0635 (17)	0.0191 (11)	0.0353 (13)	0.0096 (11)	−0.0200 (12)	−0.0077 (10)
F2	0.0424 (14)	0.0398 (13)	0.0356 (13)	0.0123 (11)	−0.0059 (11)	−0.0199 (11)
F3	0.0480 (17)	0.0369 (14)	0.151 (3)	0.0037 (12)	−0.0421 (19)	−0.0547 (19)
F4	0.089 (2)	0.0359 (14)	0.0580 (17)	−0.0372 (15)	−0.0327 (16)	0.0017 (13)
F5	0.0714 (19)	0.0366 (14)	0.0303 (13)	−0.0235 (13)	0.0121 (13)	−0.0051 (11)
F6	0.0483 (16)	0.0121 (11)	0.091 (2)	0.0016 (10)	−0.0056 (15)	−0.0042 (13)
F7	0.0314 (11)	0.0246 (10)	0.0296 (11)	−0.0078 (9)	−0.0129 (9)	−0.0115 (9)
F8	0.0269 (11)	0.0163 (9)	0.0335 (11)	−0.0055 (8)	−0.0157 (9)	0.0043 (8)
F9	0.0181 (10)	0.0226 (10)	0.0274 (10)	−0.0056 (8)	−0.0043 (8)	−0.0064 (8)
N1	0.0234 (16)	0.0277 (17)	0.048 (2)	−0.0057 (13)	−0.0110 (15)	−0.0035 (15)
O1	0.0253 (12)	0.0183 (11)	0.0200 (11)	−0.0015 (9)	−0.0096 (10)	−0.0058 (9)
O2	0.0208 (11)	0.0179 (11)	0.0163 (10)	−0.0015 (9)	−0.0071 (9)	−0.0061 (9)
O3	0.0239 (12)	0.0153 (11)	0.0217 (11)	−0.0030 (9)	−0.0080 (10)	−0.0047 (9)
O4	0.0180 (11)	0.0155 (10)	0.0165 (10)	−0.0011 (8)	−0.0058 (9)	−0.0032 (9)
O5	0.0158 (11)	0.0155 (10)	0.0185 (10)	−0.0002 (8)	−0.0074 (9)	−0.0038 (9)
O6	0.0182 (11)	0.0147 (10)	0.0200 (11)	−0.0018 (8)	−0.0094 (9)	−0.0024 (9)
O7	0.0179 (11)	0.0165 (10)	0.0178 (10)	−0.0003 (9)	−0.0062 (9)	−0.0051 (9)
O8	0.0205 (12)	0.0206 (12)	0.0213 (12)	−0.0042 (10)	−0.0002 (10)	−0.0011 (10)
S1	0.0333 (5)	0.0248 (4)	0.0270 (4)	−0.0003 (4)	−0.0169 (4)	−0.0063 (4)
S2A	0.0261 (7)	0.0370 (12)	0.0333 (8)	0.0035 (7)	−0.0148 (6)	−0.0133 (9)
C13A	0.0249 (11)	0.0239 (15)	0.0281 (13)	0.0045 (10)	−0.0145 (9)	−0.0133 (12)
C14A	0.0249 (11)	0.0239 (15)	0.0281 (13)	0.0045 (10)	−0.0145 (9)	−0.0133 (12)
C15A	0.0249 (11)	0.0239 (15)	0.0281 (13)	0.0045 (10)	−0.0145 (9)	−0.0133 (12)
C16A	0.0249 (11)	0.0239 (15)	0.0281 (13)	0.0045 (10)	−0.0145 (9)	−0.0133 (12)
S2B	0.0261 (7)	0.0370 (12)	0.0333 (8)	0.0035 (7)	−0.0148 (6)	−0.0133 (9)
C13B	0.0249 (11)	0.0239 (15)	0.0281 (13)	0.0045 (10)	−0.0145 (9)	−0.0133 (12)
C14B	0.0249 (11)	0.0239 (15)	0.0281 (13)	0.0045 (10)	−0.0145 (9)	−0.0133 (12)
C15B	0.0249 (11)	0.0239 (15)	0.0281 (13)	0.0045 (10)	−0.0145 (9)	−0.0133 (12)
C16B	0.0249 (11)	0.0239 (15)	0.0281 (13)	0.0045 (10)	−0.0145 (9)	−0.0133 (12)
S3A	0.0184 (7)	0.0314 (8)	0.0279 (10)	−0.0081 (6)	−0.0098 (7)	−0.0031 (8)
C21A	0.0154 (10)	0.0321 (12)	0.0188 (11)	0.0031 (9)	−0.0038 (9)	−0.0142 (9)
C22A	0.0154 (10)	0.0321 (12)	0.0188 (11)	0.0031 (9)	−0.0038 (9)	−0.0142 (9)
C23A	0.0154 (10)	0.0321 (12)	0.0188 (11)	0.0031 (9)	−0.0038 (9)	−0.0142 (9)
C24A	0.0154 (10)	0.0321 (12)	0.0188 (11)	0.0031 (9)	−0.0038 (9)	−0.0142 (9)
S3B	0.0184 (7)	0.0314 (8)	0.0279 (10)	−0.0081 (6)	−0.0098 (7)	−0.0031 (8)
C21B	0.0154 (10)	0.0321 (12)	0.0188 (11)	0.0031 (9)	−0.0038 (9)	−0.0142 (9)
C22B	0.0154 (10)	0.0321 (12)	0.0188 (11)	0.0031 (9)	−0.0038 (9)	−0.0142 (9)
C23B	0.0154 (10)	0.0321 (12)	0.0188 (11)	0.0031 (9)	−0.0038 (9)	−0.0142 (9)
C24B	0.0154 (10)	0.0321 (12)	0.0188 (11)	0.0031 (9)	−0.0038 (9)	−0.0142 (9)

### Geometric parameters (Å, °)

**Table:**

Nd1—O2	2.372 (2)	C19—C20	1.423 (4)
Nd1—O3	2.396 (2)	C19—H19	0.9500
Nd1—O1	2.406 (2)	C20—O6	1.256 (4)
Nd1—O6	2.411 (2)	C20—C21A	1.465 (4)
Nd1—O5	2.428 (2)	C25—N1	1.139 (5)
Nd1—O4	2.478 (2)	C25—C26	1.472 (5)
Nd1—O8	2.485 (2)	C26—H26A	0.9800
Nd1—O7	2.513 (2)	C26—H26B	0.9800
C1—F3	1.318 (4)	C26—H26C	0.9800
C1—F1	1.326 (4)	O7—H7A	0.8400
C1—F2	1.341 (4)	O7—H7B	0.8489
C1—C2	1.545 (5)	O8—H8A	0.8400
C2—O1	1.263 (4)	O8—H8B	0.8555
C2—C3	1.378 (4)	S2A—C13A	1.687 (4)
C3—C4	1.417 (4)	S2A—C16A	1.756 (7)
C3—H3	0.9500	C13A—C14A	1.455 (11)
C4—O2	1.264 (4)	C14A—C15A	1.349 (10)
C4—C5	1.467 (4)	C14A—H14A	0.9500
C5—C6	1.403 (4)	C15A—C16A	1.359 (8)
C5—S1	1.714 (3)	C15A—H15A	0.9500
C6—C7	1.423 (5)	C16A—H16A	0.9500
C6—H6	0.9500	S2B—C16B	1.754 (9)
C7—C8	1.346 (6)	C14B—C15B	1.359 (14)
C7—H7	0.9500	C14B—H14B	0.9500
C8—S1	1.709 (3)	C15B—C16B	1.330 (11)
C8—H8	0.9500	C15B—H15B	0.9500
C9—F4	1.313 (4)	C16B—H16B	0.9500
C9—F6	1.327 (4)	S3A—C24A	1.648 (7)
C9—F5	1.331 (4)	S3A—C21A	1.671 (4)
C9—C10	1.541 (5)	C21A—C22A	1.478 (10)
C10—O3	1.259 (4)	C22A—C23A	1.499 (11)
C10—C11	1.374 (5)	C22A—H22A	0.9500
C11—C12	1.403 (5)	C23A—C24A	1.369 (8)
C11—H11	0.9500	C23A—H23A	0.9500
C12—O4	1.278 (4)	C24A—H24A	0.9500
C12—C13A	1.475 (4)	S3B—C24B	1.545 (11)
C17—F8	1.336 (3)	C22B—C23B	1.484 (14)
C17—F9	1.340 (4)	C22B—H22B	0.9500
C17—F7	1.352 (3)	C23B—C24B	1.452 (12)
C17—C18	1.530 (4)	C23B—H23B	0.9500
C18—O5	1.277 (3)	C24B—H24B	0.9500
C18—C19	1.371 (4)		
			
O2—Nd1—O3	78.03 (7)	F8—C17—C18	114.9 (2)
O2—Nd1—O1	71.18 (7)	F9—C17—C18	111.6 (2)
O3—Nd1—O1	139.97 (8)	F7—C17—C18	109.5 (3)
O2—Nd1—O6	142.12 (8)	O5—C18—C19	129.7 (3)
O3—Nd1—O6	118.42 (7)	O5—C18—C17	112.3 (3)
O1—Nd1—O6	76.45 (7)	C19—C18—C17	118.0 (3)
O2—Nd1—O5	115.80 (7)	C18—C19—C20	123.6 (3)
O3—Nd1—O5	144.19 (8)	C18—C19—H19	118.2
O1—Nd1—O5	74.21 (7)	C20—C19—H19	118.2
O6—Nd1—O5	72.12 (7)	O6—C20—C19	123.9 (3)
O2—Nd1—O4	74.45 (7)	O6—C20—C21A	116.9 (3)
O3—Nd1—O4	71.76 (7)	C19—C20—C21A	119.2 (3)
O1—Nd1—O4	121.42 (7)	N1—C25—C26	178.4 (4)
O6—Nd1—O4	141.42 (7)	C25—C26—H26A	109.5
O5—Nd1—O4	80.26 (7)	C25—C26—H26B	109.5
O2—Nd1—O8	80.36 (8)	H26A—C26—H26B	109.5
O3—Nd1—O8	71.87 (8)	C25—C26—H26C	109.5
O1—Nd1—O8	78.19 (8)	H26A—C26—H26C	109.5
O6—Nd1—O8	74.15 (7)	H26B—C26—H26C	109.5
O5—Nd1—O8	140.35 (7)	C2—O1—Nd1	133.0 (2)
O4—Nd1—O8	139.12 (7)	C4—O2—Nd1	138.1 (2)
O2—Nd1—O7	144.79 (7)	C10—O3—Nd1	131.7 (2)
O3—Nd1—O7	74.20 (7)	C12—O4—Nd1	130.3 (2)
O1—Nd1—O7	142.79 (7)	C18—O5—Nd1	128.5 (2)
O6—Nd1—O7	71.78 (7)	C20—O6—Nd1	135.6 (2)
O5—Nd1—O7	77.96 (7)	Nd1—O7—H7A	109.5
O4—Nd1—O7	76.58 (7)	Nd1—O7—H7B	102.8
O8—Nd1—O7	110.36 (8)	H7A—O7—H7B	98.7
F3—C1—F1	108.7 (3)	Nd1—O8—H8A	109.5
F3—C1—F2	105.7 (3)	Nd1—O8—H8B	130.6
F1—C1—F2	105.9 (3)	H8A—O8—H8B	90.3
F3—C1—C2	111.5 (3)	C8—S1—C5	91.58 (17)
F1—C1—C2	112.6 (3)	C13A—S2A—C16A	90.3 (3)
F2—C1—C2	112.0 (3)	C14A—C13A—C12	128.0 (4)
O1—C2—C3	128.9 (3)	C14A—C13A—S2A	109.3 (4)
O1—C2—C1	114.6 (3)	C12—C13A—S2A	122.6 (3)
C3—C2—C1	116.4 (3)	C15A—C14A—C13A	115.7 (8)
C2—C3—C4	121.9 (3)	C15A—C14A—H14A	122.2
C2—C3—H3	119.1	C13A—C14A—H14A	122.2
C4—C3—H3	119.1	C14A—C15A—C16A	109.9 (8)
O2—C4—C3	124.1 (3)	C14A—C15A—H15A	125.1
O2—C4—C5	116.2 (3)	C16A—C15A—H15A	125.1
C3—C4—C5	119.7 (3)	C15A—C16A—S2A	114.4 (6)
C6—C5—C4	129.7 (3)	C15A—C16A—H16A	122.8
C6—C5—S1	111.8 (2)	S2A—C16A—H16A	122.8
C4—C5—S1	118.6 (2)	C15B—C14B—H14B	125.3
C5—C6—C7	110.5 (3)	C16B—C15B—C14B	110.4 (11)
C5—C6—H6	124.8	C16B—C15B—H15B	124.8
C7—C6—H6	124.8	C14B—C15B—H15B	124.8
C8—C7—C6	113.6 (3)	C15B—C16B—S2B	116.9 (9)
C8—C7—H7	123.2	C15B—C16B—H16B	121.6
C6—C7—H7	123.2	S2B—C16B—H16B	121.6
C7—C8—S1	112.6 (3)	C24A—S3A—C21A	93.4 (3)
C7—C8—H8	123.7	C20—C21A—C22A	129.8 (5)
S1—C8—H8	123.7	C20—C21A—S3A	118.9 (3)
F4—C9—F6	106.6 (3)	C22A—C21A—S3A	111.2 (4)
F4—C9—F5	107.2 (3)	C21A—C22A—C23A	108.2 (8)
F6—C9—F5	106.4 (3)	C21A—C22A—H22A	125.9
F4—C9—C10	111.8 (3)	C23A—C22A—H22A	125.9
F6—C9—C10	113.8 (3)	C24A—C23A—C22A	107.8 (7)
F5—C9—C10	110.6 (3)	C24A—C23A—H23A	126.1
O3—C10—C11	129.5 (3)	C22A—C23A—H23A	126.1
O3—C10—C9	112.2 (3)	C23A—C24A—S3A	116.4 (6)
C11—C10—C9	118.2 (3)	C23A—C24A—H24A	121.8
C10—C11—C12	124.3 (3)	S3A—C24A—H24A	121.8
C10—C11—H11	117.8	C23B—C22B—H22B	126.7
C12—C11—H11	117.8	C24B—C23B—C22B	106.5 (10)
O4—C12—C11	124.1 (3)	C24B—C23B—H23B	126.7
O4—C12—C13A	117.1 (3)	C22B—C23B—H23B	126.7
C11—C12—C13A	118.9 (3)	C23B—C24B—S3B	114.9 (9)
F8—C17—F9	107.2 (3)	C23B—C24B—H24B	122.6
F8—C17—F7	106.7 (2)	S3B—C24B—H24B	122.6
F9—C17—F7	106.5 (2)		
			
F3—C1—C2—O1	111.4 (4)	O6—Nd1—O3—C10	−161.9 (3)
F1—C1—C2—O1	−11.1 (5)	O5—Nd1—O3—C10	−63.1 (3)
F2—C1—C2—O1	−130.3 (3)	O4—Nd1—O3—C10	−22.7 (3)
F3—C1—C2—C3	−66.3 (4)	O8—Nd1—O3—C10	138.3 (3)
F1—C1—C2—C3	171.2 (3)	O7—Nd1—O3—C10	−103.4 (3)
F2—C1—C2—C3	52.0 (4)	C11—C12—O4—Nd1	−29.4 (4)
O1—C2—C3—C4	−4.1 (6)	C13A—C12—O4—Nd1	151.6 (2)
C1—C2—C3—C4	173.3 (3)	O2—Nd1—O4—C12	−51.4 (2)
C2—C3—C4—O2	9.0 (5)	O3—Nd1—O4—C12	30.8 (2)
C2—C3—C4—C5	−170.7 (3)	O1—Nd1—O4—C12	−107.2 (2)
O2—C4—C5—C6	−173.1 (3)	O6—Nd1—O4—C12	143.8 (2)
C3—C4—C5—C6	6.7 (5)	O5—Nd1—O4—C12	−171.8 (2)
O2—C4—C5—S1	5.2 (4)	O8—Nd1—O4—C12	2.7 (3)
C3—C4—C5—S1	−175.0 (3)	O7—Nd1—O4—C12	108.4 (2)
C4—C5—C6—C7	179.2 (3)	C19—C18—O5—Nd1	26.4 (4)
S1—C5—C6—C7	0.8 (4)	C17—C18—O5—Nd1	−153.85 (19)
C5—C6—C7—C8	0.2 (4)	O2—Nd1—O5—C18	113.3 (2)
C6—C7—C8—S1	−1.2 (4)	O3—Nd1—O5—C18	−140.7 (2)
F4—C9—C10—O3	−44.0 (4)	O1—Nd1—O5—C18	53.9 (2)
F6—C9—C10—O3	−164.8 (3)	O6—Nd1—O5—C18	−26.6 (2)
F5—C9—C10—O3	75.4 (4)	O4—Nd1—O5—C18	−179.4 (2)
F4—C9—C10—C11	137.5 (3)	O8—Nd1—O5—C18	6.3 (3)
F6—C9—C10—C11	16.7 (5)	O7—Nd1—O5—C18	−101.2 (2)
F5—C9—C10—C11	−103.1 (4)	C19—C20—O6—Nd1	−10.4 (5)
O3—C10—C11—C12	4.8 (6)	C21A—C20—O6—Nd1	170.5 (2)
C9—C10—C11—C12	−177.1 (3)	O2—Nd1—O6—C20	−88.6 (3)
C10—C11—C12—O4	4.2 (5)	O3—Nd1—O6—C20	163.3 (3)
C10—C11—C12—C13A	−176.8 (3)	O1—Nd1—O6—C20	−56.8 (3)
F8—C17—C18—O5	−166.1 (3)	O5—Nd1—O6—C20	20.7 (3)
F9—C17—C18—O5	−43.8 (3)	O4—Nd1—O6—C20	67.1 (3)
F7—C17—C18—O5	73.8 (3)	O8—Nd1—O6—C20	−138.1 (3)
F8—C17—C18—C19	13.7 (4)	O7—Nd1—O6—C20	103.6 (3)
F9—C17—C18—C19	136.0 (3)	C7—C8—S1—C5	1.4 (3)
F7—C17—C18—C19	−106.4 (3)	C6—C5—S1—C8	−1.3 (3)
O5—C18—C19—C20	−2.8 (5)	C4—C5—S1—C8	−179.8 (3)
C17—C18—C19—C20	177.4 (3)	O4—C12—C13A—C14A	8.4 (7)
C18—C19—C20—O6	−6.5 (5)	C11—C12—C13A—C14A	−170.7 (6)
C18—C19—C20—C21A	172.5 (3)	O4—C12—C13A—S2A	−169.5 (2)
C3—C2—O1—Nd1	−13.6 (5)	C11—C12—C13A—S2A	11.4 (4)
C1—C2—O1—Nd1	169.0 (2)	C16A—S2A—C13A—C14A	−5.3 (5)
O2—Nd1—O1—C2	16.8 (3)	C16A—S2A—C13A—C12	173.0 (3)
O3—Nd1—O1—C2	−24.9 (3)	C12—C13A—C14A—C15A	−173.9 (6)
O6—Nd1—O1—C2	−143.2 (3)	S2A—C13A—C14A—C15A	4.2 (9)
O5—Nd1—O1—C2	141.9 (3)	C13A—C14A—C15A—C16A	0.0 (11)
O4—Nd1—O1—C2	74.1 (3)	C14A—C15A—C16A—S2A	−4.2 (9)
O8—Nd1—O1—C2	−66.9 (3)	C13A—S2A—C16A—C15A	5.8 (5)
O7—Nd1—O1—C2	−175.1 (2)	C14B—C15B—C16B—S2B	16.0 (13)
C3—C4—O2—Nd1	4.3 (5)	O6—C20—C21A—C22A	175.0 (6)
C5—C4—O2—Nd1	−176.0 (2)	C19—C20—C21A—C22A	−4.1 (7)
O3—Nd1—O2—C4	141.2 (3)	O6—C20—C21A—S3A	−6.7 (4)
O1—Nd1—O2—C4	−12.8 (3)	C19—C20—C21A—S3A	174.2 (3)
O6—Nd1—O2—C4	19.9 (4)	C24A—S3A—C21A—C20	−178.9 (3)
O5—Nd1—O2—C4	−73.9 (3)	C24A—S3A—C21A—C22A	−0.4 (6)
O4—Nd1—O2—C4	−144.6 (3)	C20—C21A—C22A—C23A	−171.5 (4)
O8—Nd1—O2—C4	67.9 (3)	S3A—C21A—C22A—C23A	10.1 (8)
O7—Nd1—O2—C4	179.7 (3)	C21A—C22A—C23A—C24A	−17.1 (9)
C11—C10—O3—Nd1	13.3 (5)	C22A—C23A—C24A—S3A	18.2 (7)
C9—C10—O3—Nd1	−164.9 (2)	C21A—S3A—C24A—C23A	−11.0 (5)
O2—Nd1—O3—C10	54.7 (3)	C22B—C23B—C24B—S3B	−26.3 (11)
O1—Nd1—O3—C10	94.8 (3)		

### Hydrogen-bond geometry (Å, °)

**Table:**

*D*—H···*A*	*D*—H	H···*A*	*D*···*A*	*D*—H···*A*
O7—H7A···O5^i^	0.84	2.02	2.811 (3)	156
O7—H7B···O4^i^	0.85	2.06	2.812 (3)	148
O8—H8A···N1	0.84	2.15	2.902 (5)	149

Symmetry codes: (i) −*x*, −*y*+2, −*z*+1.
